
JOURNAL INFORMATION
==============================
NLM Title Abbreviation: Exp Physiol
Journal ID: Exp Physiol
NLM Journal ID: 9002940
Journal ID: EPH

Experimental Physiology

ISSN: 0958-0670
EISSN: 1469-445X
Publisher: Wiley

ARTICLE INFORMATION
==============================
PMCID: PMC12857493
DOI: 10.1113/eph.70198
Article ID: EPH70198
Article version: 1
Subjects: Issue Information, Issue Information

Issue Information

Electronic publication date: 2026 Jan 30
Publication date: 2026 Feb
Volume: 111
Issue: 2
First page: 580
Last page: 585
Copyright: © 2026 The Authors. Experimental Physiology published by John Wiley & Sons Ltd on behalf of The Physiological Society.
License: This is an open access article under the terms of the http://creativecommons.org/licenses/by/4.0/ License, which permits use, distribution and reproduction in any medium, provided the original work is properly cited.
License URL: https://creativecommons.org/licenses/by/4.0/

Figure count: 0
Table count: 0
Page count: 6

source-schema-version-number: 2.0
cover-date: 1 February 2026
details-of-publishers-convertor: Converter:WILEY_ML3GV2_TO_JATSPMC version:6.6.8 mode:remove_FC converted:30.01.2026

==============================

### Experimental Physiology

Open Access

### Integration and Translation

Volume 111 | Issue 2 | 1 February 2026

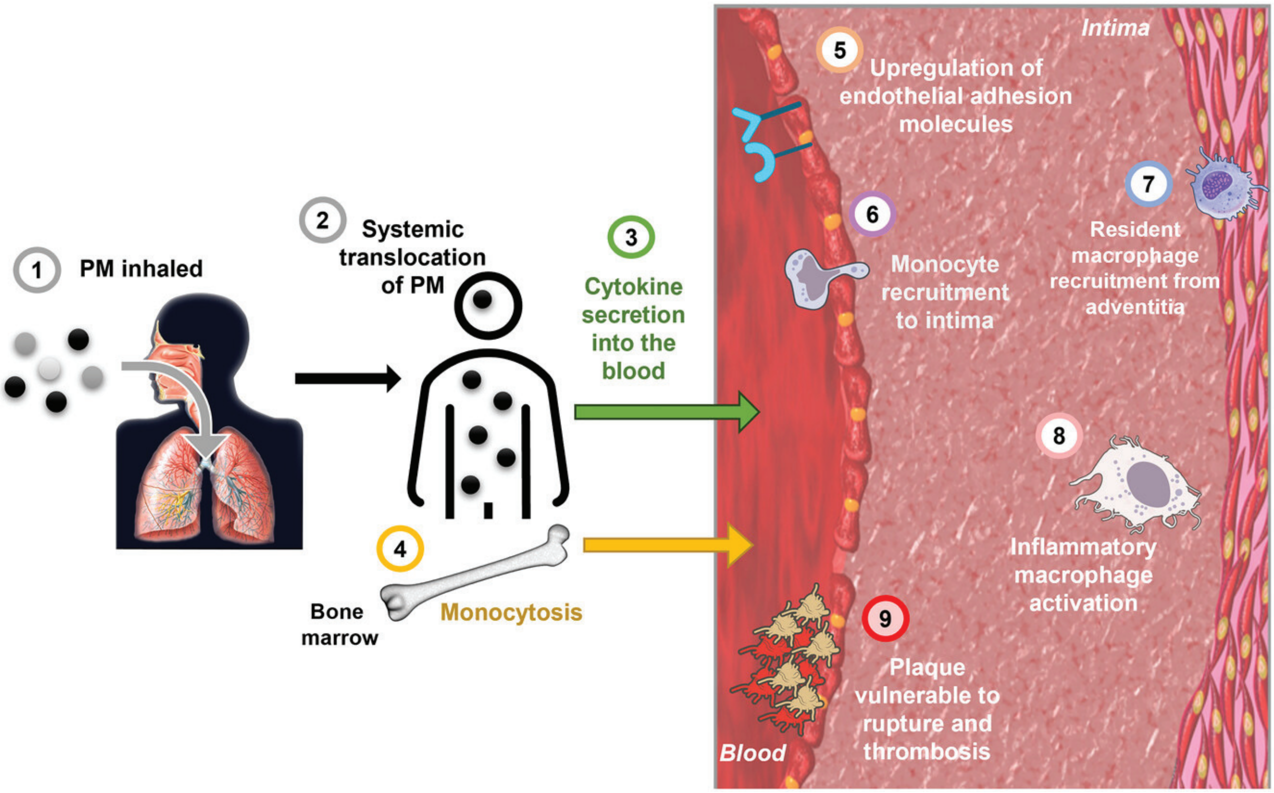

WILEY

The Physiological Society

150 YEARS 1876 2026 LEADING DISCOVERY SCIENCE

### Experimental Physiology

Open Access

Integration and Translation

Volume 111/Issue 2/
1 February 2026

### EDITORIAL BOARD

**EDITOR-IN-CHIEF**
Damian Bailey *Pontypridd, UK*

**DEPUTY EDITOR-IN-CHIEF EUROPE**
Ronan Berg *Copenhagen, Denmark*

**DEPUTY EDITOR-IN-CHIEF USA**
David Poole *Manhattan, KS, USA*

**SENIOR ETHICS EDITOR**
Susan Barman *East Lansing, MI, USA*

**STATISTICAL EDITOR**
Cody Durrer *Copenhagen, Denmark*

**SENIOR REVIEWS EDITOR**
Toby Mündel *Ontario, Canada*

**CONNECTIONS EDITOR**
Prem Kumar *Birmingham, UK*

**SENIOR EDITORS**
Dana Almohazey *Dammam, Saudi Arabia*
Jill Barnes *Madison, WI, USA*
R. Matthew Brothers *Arlington, TX, USA*
Kanokwan ‘Mandy’ Bunsawat *Iowa City, IA, USA*
Kirstene Callø *Copenhagen, Denmark*
Katrina Colafella *Victoria, Australia*
Ellen Dawson *Liverpool, UK*
Colleen Deane *Southampton, UK*
Peter Zu Eulenburg *Munich, Germany*
Federico Formenti *London, UK*
Julien Ochala *Copenhagen, Denmark*
Shigehiko Ogoh *Kawagoe, Japan*
Jason Peart *Gold Coast, Australia*
Peter Rasmussen *Copenhagen, Denmark*
Lee Romer *London, UK*
Harry B. Rossiter *Los Angeles, CA, USA*
Bill Sheel *Vancouver, Canada*
Mike Stembridge *Cardiff, UK*

**REVIEWING EDITORS**
Tatsuro Amano *Niigata, Japan*
Claire Badenhorst *Palmerston North, NZ*
Tracy Baynard *Boston, MA, USA*
Hannah Caldwell *Copenhagen, Denmark*
Havovi Chichger *Cambridge, UK*
Silvia Conde *Lisbon, Portugal*
Joseph Costello *Portsmouth, UK*
Jennifer Davies *Cardiff, UK*
Judy Delp *Tallahassee, FL, USA*
Aimee Drane *Cardiff, UK*
Esther Dupont-Versteegden *Lexington, KY, USA*
Andrea Fellet *Buenos Aires, Argentina*
Carrie Ferguson *Torrance, CA, USA*
Igor Fernandes *Niteroi, Brazil*
Ryan Hoiland *Kelowna, Canada*
Seth Holwerda *Kansas City, KS, USA*
Anna Hudson *Adelaide, Australia*
Matthew Kay *Washington, DC, USA*
James Lang *Ames, Iowa, USA*
Jacqueline Limberg *Columbia, MO, USA*
Fiona McBryde *Auckland, NZ*
Carmen De Miguel *Birmingham, AL, USA*
Niels Olesen *Copenhagen, Denmark*
Leah Reznikov *Gainesville, FL, USA*
Kleiton Silva *Camden, NJ, USA*
Sarah Short *Iowa City, IA, USA*
Nathaniel Szewczyk *Nottingham, UK*
Can Ozan Tan *Boston, MA, USA*
Mike Tymko *Vancouver, Canada*
Kai Yang *Guangzhou, China*
Song Yao *Melbourne, Australia*
Gensheng Zhang *Hangzhou, China*

**FUTURE LEADERS**
Edward Ashworth *Basel, Switzerland*
Sophie Broome *Melbourne, Australia*
Callum Brownstein *Newcastle, United Kingdom*
Daniel Craighead *Minneapolis, MN, USA*
Robert Lakin *Toronto, Canada*
Jedd Pratt *Manchester, UK*
Fabio Sarto *Padua, Italy*
Hollie Speer *Canberra, Australia*

**CONSULTANT EDITORS**
Richard Burden *Bisham, UK*
Nic Green *Bedfordshire, UK*
Julie Greeves *Salisbury, UK*
Denis Noble *Oxford, UK*
Nanduri Prabhakar *Chicago, IL, USA*

**MANAGING EDITOR**
Alex Stewart (astewart@physoc.org)

**DEPUTY MANAGING EDITOR**
Joshua Hersant (jhersant@physoc.org)

**PEER REVIEW COORDINATOR**
Diana Jones (ephjournal@physoc.org)

**EDITORIAL OFFICE**
The Physiological Society
Hodgkin Huxley House
30 Farringdon Lane, Clerkenwell
London EC1R 3AW

**PRODUCTION OFFICE**
K. Nandini (EPH@wiley.com)

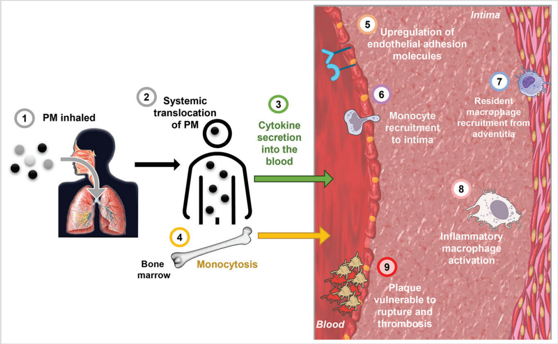

### Cover Illustration

Potential mechanisms by which pollutants such as particulate matter cause vascular dysfunction and promote atherosclerosis. See Seneviratne et al. Pages 321–334.

### Open Access and Copyright

All articles published by Experimental Physiology [submitted after 18 October 2022] are fully open access: immediately freely available to read, download, and share. All articles [accepted from 18 October 2022] are published under the terms of a Creative Commons license.
Copyright on any research article published by Experimental Physiology is retained by the author(s).
Further information about open access licenses and copyright can be found at: https://authorservices.wiley.com/author-resources/Journal-Authors/licensing/licensing-info-faqs.html#4. Information on the licenses used by Experimental Physiology can be found at: https://physoc.onlinelibrary.wiley.com/journal/1469445X.

For submission instructions and all other information visit: https://physoc.onlinelibrary.wiley.com/journal/1469445X

The Physiological Society and The Physiological Society logo are trade marks belonging to The Physiological Society and are registered in the UK and in the EU Community, respectively.

*Experimental Physiology* publishes high quality, original, physiological research papers that give novel insights into fundamental homeostatic and adaptive responses in health and papers that further our knowledge of pathophysiological mechanisms in diseases.

Experimental Physiology
Open Access
Integration and Translation

### INFORMATION AND GUIDANCE FOR AUTHORS

### Full version online at http://ep.msubmit.net/cgi-bin/main.plex?form_type=display_requirements

### ORIGIN

*The Quarterly Journal of Experimental Physiology*, founded in 1908 by Sir Edward Sharpey-Schafer, Professor of Physiology, University of Edinburgh, became a publication of The Physiological Society in 1981. The title was changed to *Experimental Physiology* in 1990.

### SCOPE

*Experimental Physiology* publishes research papers that report novel insights into homeostatic and adaptive responses in health, as well as those that further our understanding of pathophysiological mechanisms in disease. We encourage papers that embrace the journal's orientation of translation and integration, including studies of the adaptive responses to exercise, acute and chronic environmental stressors, growth and aging, and diseases where integrative homeostatic mechanisms play a key role in the response to and evolution of the disease process. Examples of such diseases include hypertension, heart failure, hypoxic lung disease, endocrine and neurological disorders. We are also keen to publish research that has a translational aspect or clinical application. Comparative physiology work that can be applied to aid the understanding human physiology is also encouraged.

Manuscripts that report the use of bioinformatic, genomic, molecular, proteomic and cellular techniques to provide novel insights into integrative physiological and pathophysiological mechanisms are welcomed.

We are also interested in methodological papers reporting important new developments in physiological techniques that could lead to significant new insights.

Papers which observe a significant new physiological behaviour or phenomenon which has the potential to alter the understanding of that field, but which have not yet identified the mechanisms may also be considered. Such papers should demonstrate the experimental examination and exclusion of a number of plausible mechanisms for the important novel phenomenon.

### Article Types

In addition to original research, *Experimental Physiology* consider the following article types for publication:

*Case studies, Short Communication, Connections, Reviews, Full Reviews, Hot Topic Reviews Myths and Methodologies and Symposium Reports.*

Review articles are normally by invitation but proposals may be submitted for consideration.

Commentary articles in the form of Viewpoints and Letters are also published.

For full guidelines on all published article see full guidelines.

C O P E COMMITTEE ON PUBLICATION ETHICS

This journal is a member of and subscribes to the principles of the Committee on Publication Ethics

### ETHICS OF EXPERIMENTATION

**Animal Experiments** - see full journal policy at https://physoc.onlinelibrary.wiley.com/hub/animal-experiments

Experimental Physiology endorses the ARRIVE Guidelines for reporting *in vivo* experiments: http://onlinelibrary.wiley.com/doi/10.1113/expphysiol.2010.053793/full

Authors of papers submitted to Experimental Physiology must adhere to the journals' principles and standards for reporting animal experiments.

Authors should read these guidelines to ensure that their research and experiments adhere to our exacting standards.

Euthanasia/humane killing: Animals must be killed using methods approved for that species, stage of development and size. The standards required by the journals are those of the UK/European Union legislation, set out in in ASPA Schedule 1 in the UK and in Annex IV in the European Directive 2010/63/EU. Methods not on Schedule 1 or Annex IV could be permissible but even where the method used conforms with national guidelines, the onus is on the author to provide scientific evidence to demonstrate that it is at least as humane as those on Schedule 1 and Annex IV.

Our guidelines have been outlined in the Editorial 'Principles and standards for reporting animal experiments in Experimental Physiology'.

**Confirmation of compliance**

Authors must state in the Methods section that the investigators understand the ethical principles under which the journal operates and that their work complies with the animal ethics checklist as outlined in the Editorial.

**Human Experiments** - See full journal policy at https://physoc.onlinelibrary.wiley.com/hub/human-experiments

The Editorial Boards are concerned that all work published in The Journal of Physiology and Experimental Physiology should have been conducted according to the highest standards of safety and ethics. Legislation and accepted practice concerning human experimentation vary from country to country and consequently it is difficult to define absolute requirements. However, work with human subjects should conform to the standards set by the Declaration of Helsinki (last modified in 2013), the Medical Research Council's online guidance on the use of human tissue, and the guidelines set out below. The objectives must be to ensure that all risks are minimized and that subjects are not injured and do not feel they have been abused as a result of participating in the study. Any definition of abuse will include excessive or unexpected pain or discomfort experienced during the experiment. Note, in the case of experiments involving minors, any risks must have been considered absent or minimal, and evidence must be presented that the experiments were performed with the understanding and consent of the legal guardian.

All manuscripts must contain statements indicating that (i) informed consent has been obtained, preferably in writing, (ii) studies conformed to the standards set by the Declaration of Helsinki or the version that was in place at the time of the experiments, NOTE: If the research study was registered (clause 35 of the Declaration of Helsinki) the registration database should be indicated, else the lack of registration should be noted as an exception (e.g. The study conformed to the standards set by the Declaration of Helsinki, except for registration in a database.); and (iii) the procedures have been approved by the local ethics committee.

### DISCLAIMER

The Publisher, the Physiological Society and Editors cannot be held responsible for any errors in or any consequences arising from the use of information contained in this journal. The views and opinions expressed do not necessarily reflect those of the Publisher, the Physiological Society or Editors, neither does the publication of advertisements constitute any endorsement by the Publisher, the Physiological Society, Editors, or Authors of the products advertised.

Wiley Open Access articles posted to repositories or websites are without warranty from Wiley of any kind, either express or implied, including, but not limited to, warranties of merchantability, fitness for a particular purpose, or non-infringement. To the fullest extent permitted by law Wiley disclaims all liability for any loss or damage arising out of, or in connection with, the use of or inability to use the content.

Experimental Physiology

Vol 111.2

### Contents of volume 111.2

1 February 2026

### REVIEW ARTICLE

### 2024 INAUGURAL REVIEW PRIZE WINNER

321 Environmental modulators of vascular physiology and inflammation
*Anusha N. Seneviratne, Anne Majumdar, Kalpana Surendranath and Mark R. Miller*

### EDITORIAL

335 Caffeine in cerebrovascular research: To withdraw or not to withdraw?
*Brooke R. Shepley, Kevin J. Milne and Anthony Richard Bain*

340 A shaking experience: My exposure to hypothermia
*Ryan P. Sixtus*

### VIEWPOINT

344 From fatigue to physiology: Submaximal 2-day cardiopulmonary exercise test and emerging standards in long COVID
*Michael G. Risbano*

346 Cardiac vagal activity is not a determinant of apnoea tolerance in trained breath-hold divers
*Benjamin S. Stacey and Giorgio Manferdelli*

### REVIEW ARTICLE

349 Are biologically meaningful effect sizes a factor in study design? A systematic review of translational chronic variable stress studies
*Crispin Jordan, Nicola Romanò and John Menzies*

### RESEARCH ARTICLE

### CARDIOVASCULAR CONTROL

357 Quantification of the cerebral pressure–flow relationship directional sensitivity: Reliability of shorter repeated squat–stand durations
*Lawrence Labrecque, Marc-Antoine Roy, Shahrzad Soleimani Dehnavi, Mahmoudreza Taghizadeh, Joel S. Burma, Jonathan D. Smirl and Patrice Brassard*

375 Effects of 12 weeks of upper-body rowing exercise on autonomic cardiovascular control and vascular structure in spinal cord-injured humans
*Rasmus Kopp Hansen, Rasmus Bering, Claus Graff, Stefanos Volianitis, Uffe Laessoe, Afshin Samani and Ryan Godsk Larsen*

### ENDOCRINOLOGY AND METABOLISM

390 A comparison of methods to predict ovulation day, menstrual cycle characteristics and variability in professional female soccer players
*Rosie Anderson, Ian Rollo, Daniel Martin, Richard Burden, Rebecca K. Randell, Craig Twist and Samantha L. Moss*

### ENVIRONMENTAL AND EXERCISE

403 Faecal microbiome, gastrointestinal integrity, inflammation and thermoregulation in recent exertional heat illness patients and matched controls
*Alex A. M. Gould, Neil P. Walsh, Michael J. Tipton, Michael J. Zurawlew, Omar Tayari, Carol House, Simon K. Delves, Samuel C. Robson, Janis J. Shute, Joy E. M. Watts, Andrew J. Roberts, Alex J. Rawcliffe, Megan R. Robinson and Jo Corbett*

426 Skin wetness perception across body sites in children and adolescents aged 7–16 years old
*Alessandro Valenza, Hannah Blount, Jade Ward, Charlotte Merrick, Riley Wootten, Jasmin Dearden, Charlotte Wildgoose, Antonino Bianco, Alex Buoite-Stella, Victoria L. Filingeri, Peter R. Worsley and Davide Filingeri*

435 Submaximal 2-day cardiopulmonary exercise testing to assess exercise capacity and post-exertional symptom exacerbation in people with long COVID
*Callum Thomas, Nik Kudiersky, Paul Ansdell, Ruth E Ashton, Calum Brown, Thomas Bewick, Jack Carr, Emily Hume, Padraig Spillane, Elisa Pastorio, Rebecca Owen, Tom Maden-Wilkinson, Ethan McNeil-Angopa, Tom Parkington, Ross Arena, Cemal Ozemek, Federico Formenti, Sundar Kumar Veluswamy, Rachita Gururaj and Mark A. Faghy*

449 Effect of prolonged voluntary wheel running on oxidative stress and defence mechanisms in cortex and hippocampus of healthy female rats
*Camilla Myrup Holst, Iria Esperon-Abril, Frederik Bryske Juhl, Jesper Emil Jakobsgaard, Jonas B. Kristiansen, Kristian Vissing and Tinna Stevnsner*

### MUSCLE

464 Muscle oxygen saturation plateau: Definition and verification in different oxygen availability conditions in locomotor and non-locomotor muscles
*Tomasz Kowalski, Kinga Rębiś, Adrian Wilk, Dominika Granda, Andrzej Białecki, Tadej Debevec and Raphael Faiss*

474 Effects of soluble guanylyl cyclase stimulation on muscle oxygenation and exercise capacity in heart failure with mildly reduced ejection fraction
*Ramona E. Weber, Kiana M. Schulze, Andrew G. Horn, Tyler E. McCoach, Zachary J. White, K. Sue Hageman, Stephanie E. Hall, Peter Sandner, Brad J. Behnke, Timothy I. Musch and David C. Poole*

### NEUROSCIENCE

489 Neuroprotective effects of sinomenine and metformin in diabetic stroke: Role of NLRP3/caspase-1 and mitophagy
*Wendi Li, Yiying Fan and Shaik Althaf Hussain*

501 Microelectrode recordings from the human cervical vagus nerve during maximal breath-holds
*Vaughan G. Macefield, Anthony R. Bain, Matthew I. Badour, Marko Kumric, Ivan Drvis, Otto F. Barak, Josko Bozic and Zeljko Dujic*

### RENAL

517 Empagliflozin does not prevent progression of Dent's disease type 1 in a mouse model
*Elise de Combiens, Nadia Frachon, Yohan Bignon, Marc Fila, Clément Brossard, Perrine Frère and Stéphane Lourdel*

### RESPIRATORY

527 Effect of moderate altitude and nocturnal oxygen therapy on cerebrovascular function in patients with COPD: A randomized, crossover trial at 2048 m
*Dominic Gilliand, Tsogyal D. Latshang, Sayaka S. Aeschbacher, Fabienne Huber, Deborah Flueck, Mona Lichtblau, Stefanie Ulrich, Elisabeth D. Hasler, Philipp M. Scheiwiller, Julian Müller, Silvia Ulrich, Konrad E. Bloch and Michael Furian*

539 Clinical significance and immune microenvironment association of cuproptosis-related genes in pan-cancer
*Xinyu Ge, Kaijing Wang, Tian Zhao, Jinyi Wang, Jie Liu, Zhengliang Sun, Zhengjun Chai, Wen Zhang, Chengbao Li, Yan Xu and Guohan Chen*

556 Alveolar deadspace and intrapulmonary shunt in healthy individuals and in individuals who have recovered from COVID-19 infection
*Dominic Sandhu, Snapper R. M. Magor-Elliott, Nayia Petousi, Nick P. Talbot, Alexander N. Bennett, David A. Holdsworth, Grant A. D. Ritchie and Peter A. Robbins*

### VASCULAR

568 Sex differences in cerebral pulsatility and damping: A 4D flow MRI study
*Sarean Harmoni A. Gaynor-Metzinger, Alexander M. Norby, Brandon G. Fico, M. Erin Moir, Nicole A. Loggie, Kathleen B. Miller, Adam T. Corkery, Andrew G. Pearson, Anna J. Howery, Leonardo A. Rivera-Rivera, Kevin M. Johnson, Sterling C. Johnson, Oliver Wieben, Ryan D. Zea and Jill N. Barnes*

Editor's Pick

Potential mechanisms by which pollutants such as particulate matter cause vascular dysfunction and promote atherosclerosis. See Seneviratne et al. Pages 321–334.

Reasons to submit to

### Experimental Physiology

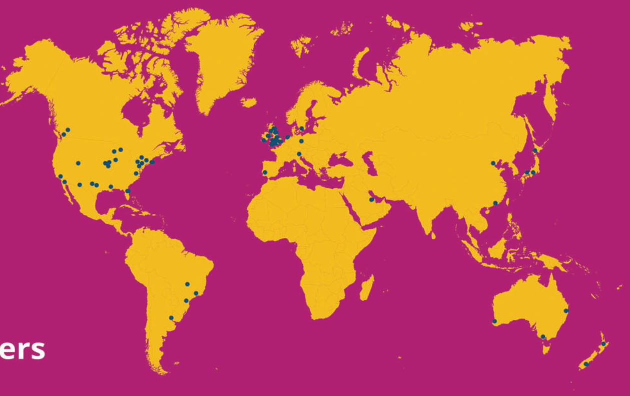

**Annual prizes for early and mid-career researchers**

**68 Board Members from 14 counties**

Published research from **24** countries in 2024

**Publishes articles in all areas in physiology.** Publishes novel insights into homeostatic and adaptive responses in health and disease.

OPEN

**Get your work seen, read and cited with Open Access publication.**

8 years

Cited half-life of over 8 years

Over 1 million downloads per year

32

An average of 32 days from submission to first decision with two reports

Acceptance to online publication within 5 days

No page or figure limits

Pre-submission enquiries encouraged ephjournal@physoc.org

Prior publication on preprint servers allowed

1/3

46% of articles accepted in 2024

14 Nobel Prize winners

**2.6**
2023 Impact Factor

Editor-in-Chief
**Damian Bailey**
University of South Wales, UK

**ep.physoc.org**

**bit.ly/ExpPhysiolLinkedIn**

**ephjournal@physoc.org**

**/expphysiol**

**@ExpPhysiol**

WILEY
